# Network Analysis of Global Influenza Spread

**DOI:** 10.1371/journal.pcbi.1001005

**Published:** 2010-11-18

**Authors:** Joseph Chan, Antony Holmes, Raul Rabadan

**Affiliations:** Department of Biomedical Informatics and Center for Computational Biology and Bioinformatics, Columbia University College of Physicians and Surgeons, New York, New York, United States of America; University of California San Diego, United States of America

## Abstract

Although vaccines pose the best means of preventing influenza infection, strain selection and optimal implementation remain difficult due to antigenic drift and a lack of understanding global spread. Detecting viral movement by sequence analysis is complicated by skewed geographic and seasonal distributions in viral isolates. We propose a probabilistic method that accounts for sampling bias through spatiotemporal clustering and modeling regional and seasonal transmission as a binomial process. Analysis of H3N2 not only confirmed East-Southeast Asia as a source of new seasonal variants, but also increased the resolution of observed transmission to a country level. H1N1 data revealed similar viral spread from the tropics. Network analysis suggested China and Hong Kong as the origins of new seasonal H3N2 strains and the United States as a region where increased vaccination would maximally disrupt global spread of the virus. These techniques provide a promising methodology for the analysis of any seasonal virus, as well as for the continued surveillance of influenza.

## Author Summary

As evidenced by several historic vaccine failures, the design and implementation of the influenza vaccine remains an imperfect science. The virus's rapid rate of evolution makes the selection of representative strains for vaccine composition a difficult process. From a global health viewpoint, how to optimally implement a limited stockpile of vaccines is another fundamental question that remains unanswered. An understanding of how influenza spreads around the world would greatly aid the design and implementation process, but regional and seasonal bias in collected virus samples hampers epidemiologic analysis. Here, we show that it is possible to counter this data bias through probabilistic modeling and represent the global viral spread as a network of seeding events between different regions of the world. On a local scale, our technique can output the most likely origins of a virus circulating in a given location. On a global scale, we can pinpoint regions of the world that would maximally disrupt viral transmission with an increase in vaccine implementation. We demonstrate our method on seasonal H3N2 and H1N1 and foresee similar application to other seasonal viruses, including swine-origin H1N1, once more seasonal data is collected.

## Introduction

Influenza, a negative-sense RNA orthomyxovirus, is one of the few diseases that is truly global in scale. It is responsible for approximately three to five million cases of severe acute respiratory illness and 250,000 to 500,000 deaths each year throughout the world [1]. In 2009, the swift isolation of swine-origin H1N1 strain (S-OIV) from all continents within several weeks of onset reinforced the idea that influenza is a highly infectious agent circulating worldwide [2], [3].

Although vaccination remains one of the most powerful ways of combating influenza, choosing a representative strain for vaccine composition poses a challenging problem. Due to the virus's high evolutionary rate, significant resources must be spent to update vaccines each year in order to match the dominant epitope of the season. Even with annual strain selection, major antigenic reassortment can obviate otherwise promising vaccine candidates, as occurred with the ‘Fujian/411/2002’-like H3N2 strain in 2003 [4], [5]. To prevent such vaccine failures, a solid understanding of the global spread of influenza must inform the design process. If reservoirs for new viral strains can be identified, surveillance in these areas can better optimize prediction of seasonal variants in seeded regions.

Previous papers investigating the global circulation of H3N2, the major seasonal influenza subtype prior to pandemic H1N1, focused on transmission within and between climate zones. Important motivating factors for such analysis include increased aerosol transmission in cold and dry conditions, as well as increased indoor crowding and decreased host immunity in cold and wet conditions [6], [7]. In the temperate zones, influenza exhibits distinct seasonality with flu-related cases spiking in the winter. However, several papers have confirmed the presence of viral diversity even between these epidemic peaks [8], [9], [10], suggesting two possible scenarios during the inter-epidemic period: either viral infections locally persist at a low level only to reemerge as the dominant strains of the epidemic season, or an outside source introduces new genetic diversity into temperate populations each year. Although a degree of local persistence may occur, phylogenetic analysis supports the latter scenario, with few direct links between strains of the same region but successive seasons [8], [9], [10].

For a given temperate zone, these conclusions suggest the tropics or the opposite temperate zone as plausible external seeding regions. At first blush, northern-southern temperate oscillations seem credible. Each year, northern and southern temperate climates have alternating seasonal influenza epidemics, lasting from November to April, and May to September respectively [11]. A possible mechanism of viral spread could involve transmission from the seasonal peak of one temperate zone into the season ebb of the other. On the other hand, specific epidemiological characteristics suggest a tropical origin for influenza. For example, although both climates share a similar yearly burden of mortality from influenza, the tropics do not possess the same consistent seasonal peaks during the winter months [9], [12], [13]. With a constant, low-level circulation of viruses year-round, the tropics represent an ideal epicenter for the extended transmission of new viruses to the rest of the world [14], [15], [16].

Several papers tracking H3N2 across continents have asserted that this tropical reservoir of influenza strains lies within East-Southeast Asia [12], [14], [17]. Russell, et al. analyzed H3N2 data to identify regions of the world that are antigenically and genetically leading or trailing. They found that newly emerging strains appeared in E-SE Asia roughly 6–9 months earlier than in other parts of the world, while South America experienced delayed transmission of roughly 6–9 months following other parts of the world [8].

However, such studies have been limited by several drawbacks. Most papers focus on H3N2 as a single entity, when in reality, it co-circulates with several other subtypes, the most important of which is seasonal H1N1 [11]. Although they possess different surface antigens, H3N2 and H1N1 share enough genetic similarity to display cross-immunity. As a result, seasonal H1N1 may demonstrate transmission patterns distinct from H3N2's [18], [19]. Such codependence between different subtypes is exemplified by the pandemic years of 1957 and 1968, when H2N2 replaced preexisting H1N1 and H3N2 replaced preexisting H2N2, respectively [20], [21]. Similarly, the antigenically different pandemic H1N1 strain of 2009 has largely overtaken previously circulating H1N1 and H3N2 [22]. During the years our dataset took place, evidence that H3N2 and H1N1 rarely co-dominate in a season further supports the idea of codependent dynamics [7].

A second shortcoming stems from biases in the number of sequences from different regions and different seasons [8]. Most isolates of H3N2 and H1N1 were sampled from North America, whereas Africa and South America have been largely neglected [23]. Many sequences were obtained within the last 15 years, making reliable tracking over long periods of time problematic. On the level of climate zones, the number of temperate isolates far outstrips the tropics. Although hemagglutinin (HA), the HA1 domain, and neuraminidase (NA) have the most globally representative distributions of sequences, even these remain skewed (Figure S1, Figure S2).

**Figure 1:**
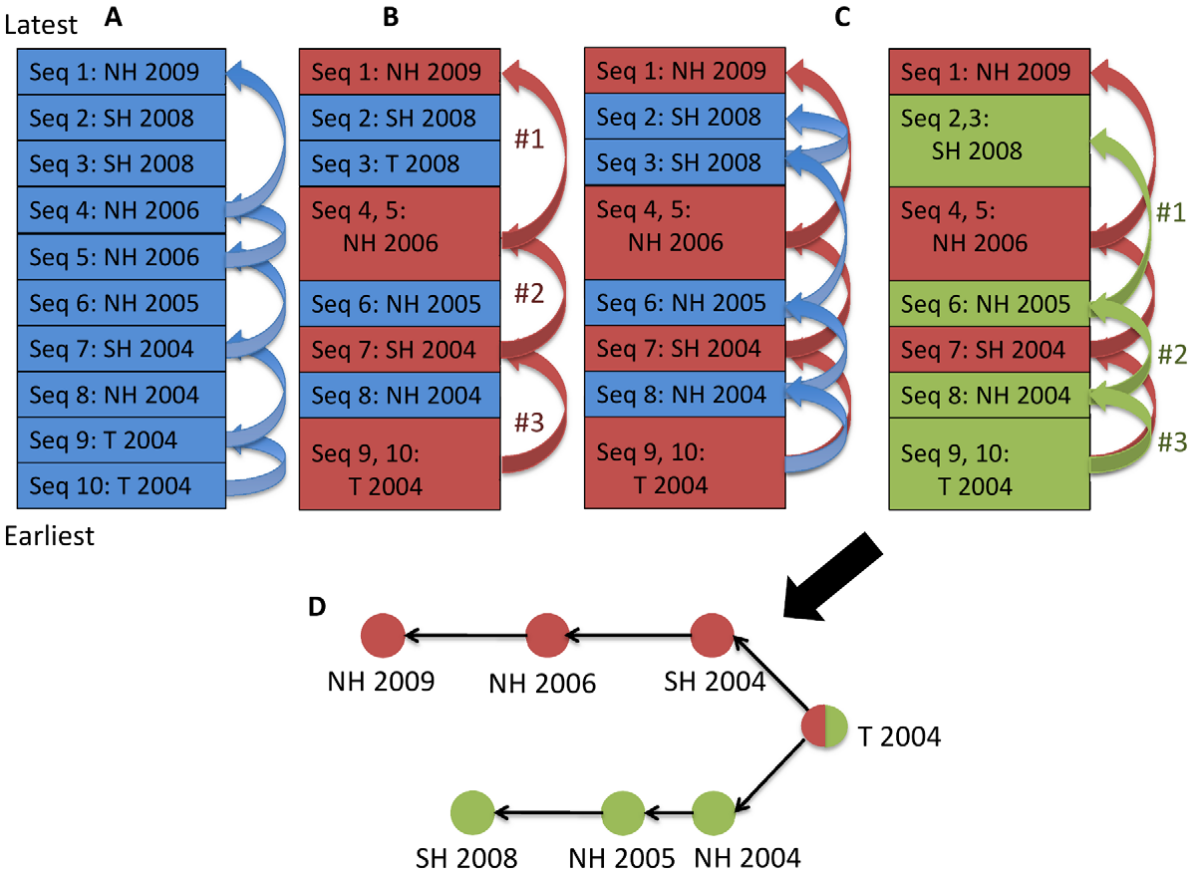
Methodology for spatiotemporal clustering. (A) We first ordered sequences from NCBI from earliest to latest. Starting with the most recent virus “Seq 1,” we worked backwards, tracing the most parsimonious evolutionary path of the virus until we reached the oldest sequence “Seq 10.” To accomplish this goal, we defined each virus's most likely ancestor to have the highest sequence similarity among all older viruses. (B) Contiguous sequences along the evolutionary path were clustered (grouped) together by common geography and season. (C) The process was repeated starting with the next most recent virus not yet included in the evolutionary path. (D) The cycle continued until all sequences were connected in a tree of clustered evolutionary paths.

**Figure 2:**
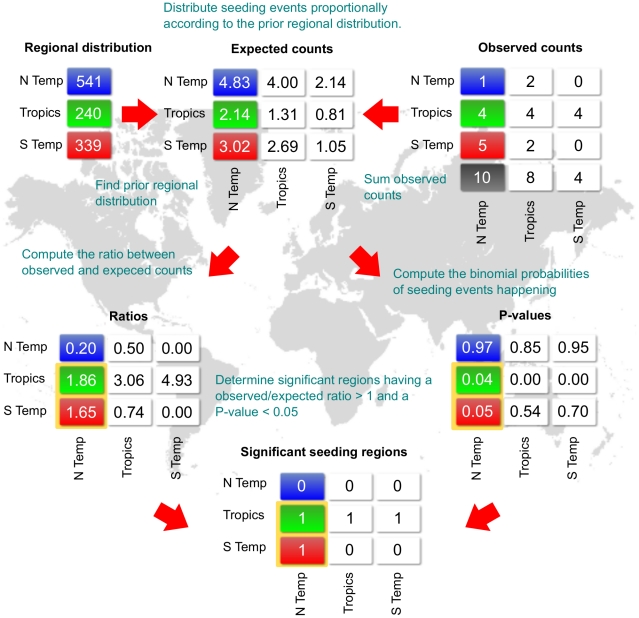
Methodology for determining significant seasons. As an example, consider the 2003-04 flu season. After clustering, there were a total of 10 observed seeding events into the northern temperate zone: 1 from the north, 4 from the tropics, and 5 from the south. Up until that year, the skewed regional distribution of HA sequences included 541 (48.3%) northern temperate, 240 (21.4%) tropical, and 339 (30.2%) southern temperate isolates. Multiplying these percentages with the 10 observed seeding events yielded expected counts of 4.8, 2.1, and 3.0. Therefore, the number of seeding events from the north was less than expected, and from the tropics and the south, more than expected. Corresponding binomial p-values—0.986, 0.043, and 0.049, respectively—indicated that there were two statistically significant events, the most significant of which was transmission from the tropics into the northern temperate zone. Similar analysis for transmission into the tropics and the southern temperate showed that only the tropical zone was a significant seeder.

In this paper, we present a novel probabilistic model for tracking the spread of influenza that employs two strategies to eliminate regional and seasonal data bias. The first involves clustering isolates of high sequence similarity by region and season. Since we would expect highly similar sequences from the same time and location to be related, we considered seeding events between clusters to be of greater significance. Consideration of clusters rather than individual sequences nullifies the over-representation of a high number of isolates from a single region and season (Figure 1). As a second strategy for eliminating bias, we determined statistical significance of inter-cluster seeding events by modeling transmission as a binomial distribution with prior probabilities based on the proportion of sequences isolated before a given time point. To illustrate our methodology, Figure 2 depicts the 2003–2004 flu season, which was marked by failure to predict the dominant, tropically-derived Fujian/411/2002-like H3N2 strain. We identified a strong seeding pattern from the tropics to all three climate zones, supporting the effectiveness of our methodology.

We applied this model to the H3N2 and H1N1 coding regions of HA and NA, the most antigenic proteins of the eight viral segments. Clustering H3N2 sequences confirmed previous findings that this strain originates in the tropics, specifically E-SE Asia, and seeds South America by way of North America last. Clustering H1N1 NA also revealed a similar pattern of circulation beginning in the tropics. However, similar H1N1 analysis by continent and country was not possible due to the absence of a larger number of countries in the dataset.

Applying the same methodology to the H3N2 HA1 domain increased the geographic diversity enough to enable reconstruction of the global influenza network prior to the 2009 pandemic strain at a country level. Our results suggest a possible flu seeding hierarchy beginning in China and spreading throughout a highly interconnected E-SE Asian subnetwork. From there, viruses transmit to an Oceanic subnetwork dominated by interchange between Australia and New Zealand. Both subnetworks seed into the USA, which in turn seeds many countries, particularly in South America.

Expanding upon the sink-source hypothesis of global influenza dynamics proposed by Rambaut, et al. [15], we applied techniques of graph theory to identify important source and sink regions in the global flu network. These techniques better describe the dynamic nature of influenza movement across the globe, as well as suggest different vaccination strategies to disrupt maximally viral flow around the world.

## Results

### Emergent Strains from the Tropics and Asia

Spatiotemporally clustering the complete H3N2 and H1N1 coding sequences for HA and NA allowed the determination of multiple statistically significant seeding seasons between 1988 and 2009. For our initial analysis, we clustered sequences into three climate zones—northern temperate, tropical, and southern temperate. To determine seasonal boundaries, we defined the northern temperate season to last from 1^st^ July to the 30^th^ June of the following year and the southern temperate season to last from 1^st^ January to the 31^st^ December of the same year [11]. Although the tropics do not have a well-defined seasonal pattern, we determined a consensus tropical flu season from 1^st^ October to 30^th^ September of the next year (Text S1, Table S1).

**Figure 3:**
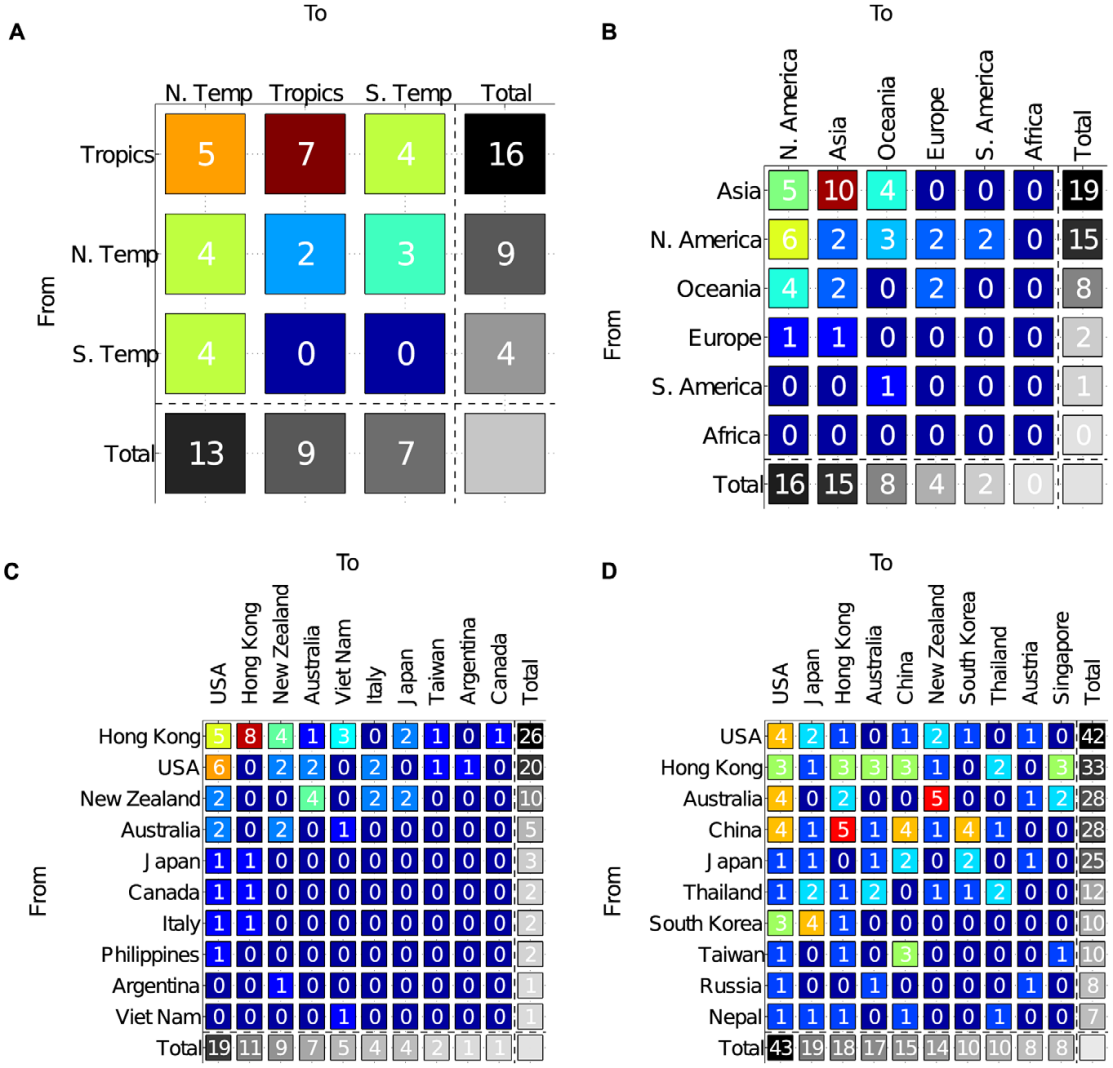
Clustering the complete NA coding sequences of H3N2 by (A) climate zone, (B) continent, and (C) country. Top H3N2 seeders were located in the E-SE Asian tropics, particularly in Hong Kong. (D) Clustering the H3N2 HA1 domain by country increased the total number of sequences and countries under consideration. This analysis identified USA, Hong Kong, Australia, and China as the top H3N2 seeders in that order. For all country heat maps, only countries transmitting or receiving at least one significant seeding season were included. For each entry, there was a maximum number of 22 seeding seasons, the range in the date of isolation for all datasets.

**Figure 4:**
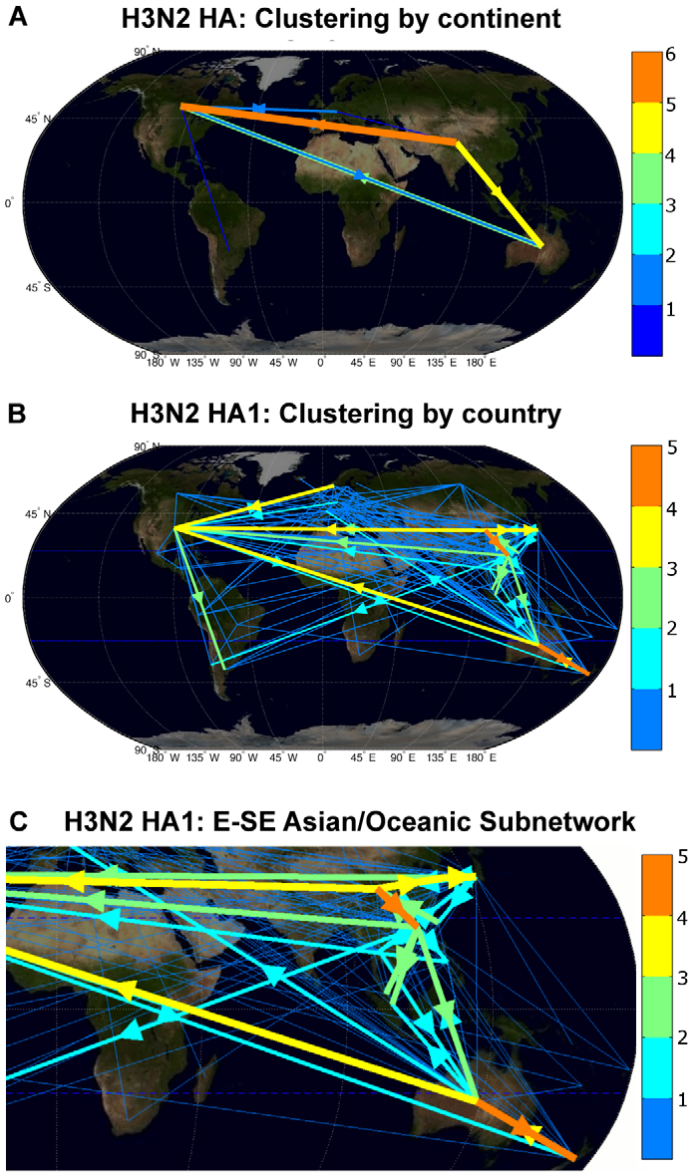
Global network of statistically significant seeding seasons for H3N2 after clustering by (A) continent and (B) country. (A) Seasonal variants emerge from Asia and make their way to North America. A smaller connection from North America to South America is consistent with the finding that South American isolates are antigenically delayed [8]. (B) Clustering by country showed tropic-centric movement patterns. (C) H3N2 seeding events in the South-East Asian/Oceanic subnetwork showing China, Hong Kong, and Australia as major hubs. Arrows signify the direction of the seeding event. Each edge is color-coded according to weight: the number of seeding events represented. For visual simplicity, arrowheads were omitted for edges of unit weight. Edges connect between the centroids of two continents or countries. World map image taken from: onearth.jpl.nasa.gov.

Results for H3N2 showed that the overwhelming majority of statistically significant seeding seasons came from the tropics, confirming previous findings (Figure 3A, Figure S3A). Clustering H3N2 by the six major continents rendered an even more detailed picture. For HA, Asia was the primary seeder of Asia, North America, and Oceania. Prominent transmission from North America to Europe and South America was also observed (Figure S3B). Interestingly, this hierarchical seeding structure reflects the findings of Russell, et al., which identified Asia and South America as antigenically advanced and lagging continents respectively [8]. This network of hierarchical seeding can be visualized as a directed graph plotted against the world map (Figure 4A). Analysis of NA produced similar findings with the exception of North America being its own primary seeder (Figure 3B). No complete HA and NA isolates existed in the NCBI Influenza Virus Resource database [24] for Africa.

The complete dataset of HA and NA represented only 17 and 21 countries respectively. Despite the sparse number of countries for analysis, both HA (Figure S3C) and NA (Figure 3C) consistently identified Hong Kong (considered a country by NCBI sequence annotation) as the primary external seeder of USA and New Zealand among others, and New Zealand as the primary external seeder of Australia.

Due to fewer available sequences, clustering H1N1 did not yield as many significant seeding events as H3N2; however, our tests suggest that H1N1 adopts a similar seeding pattern with the tropics as a source. Of the two segments, NA sequences display a broader geographical profile than HA. In particular, our HA dataset for H1N1 contained no sequences from Hong Kong and only 1 (0.091%) China sequence, while NA contained 9 (0.69%) Hong Kong and 3 (0.23%) China sequences. Consequently, we considered NA to be more suitable for comparison between H3N2 and H1N1 and HA to be a background signal to assess the effect of Hong Kong and China on global influenza transmission. Even so, the number of these H1N1 Hong Kong and China sequences remained vastly disproportionate to the 361 (7.42%) Hong Kong and 133 (2.73%) China sequences of H3N2.

**Figure 5:**
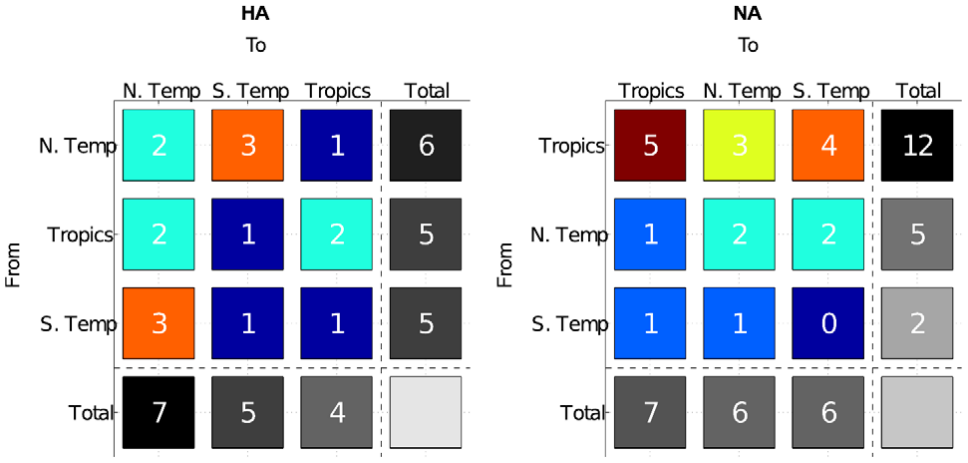
Clustering the H1N1 (A) HA and (B) NA segments by climate. HA counts were noticeably low and homogeneous, compared to NA counts that reflected a strong signal from the tropics. One explanation is the lack of Hong Kong and China sequences in HA compared to NA. The difference between HA and NA counts may reflect the impact of including even a marginal number of Hong Kong and China sequences. If H1N1 sequences were more evenly distributed by region, one may anticipate seeding counts more aligned with those of H3N2. For each entry, there was a maximum number of 22 seeding seasons, the range in the date of isolation for all datasets.

Clustering H1N1 NA by climate zone supported the theory of global viral spread from the tropics (Figure 5B). Unlike H3N2, H1N1 analysis by continent and country was inconclusive due to low (typically fewer than 3 seeding events), homogeneous counts. Although inconclusive, the fact that a tropical signal could be detected at all from such few tropical countries, including Hong Kong and China, suggests that H1N1 adopts a similar seeding pattern out of the tropics. Due to insufficient sampling, however, a more detailed transmission pattern could not be discerned.

### The Global Seeding Network of H3N2 by Country

Although using the complete HA and NA coding genomes facilitated differentiation of isolates by Hamming distance, the absence of data from certain countries limited the information gained from clustering at this geographic detail, a problem that has plagued previous studies [8]. To increase the amount of data from different geographical regions, we clustered H3N2 sequences of the HA1 epitope, expanding the number of isolates in the dataset from 2,251 to 4,864, and the number of countries from 17 to 81. A necessary consequence of expanding geographic coverage was an increase in the number of non-unique solutions (Text S1).

Importantly, clustering HA1 by climate and continent was corroborated by findings from the complete HA and NA sequences, lending credence to the validity of the dataset. Due to the inclusion of isolates from Africa, which was hitherto not present in our datasets, H3N2 HA1 analysis also revealed Europe and North America tied for being the primary seeders of Africa.

Country clustering of the HA1 data produced a highly detailed global network of influenza variants. USA, Hong Kong, Australia, and China were identified as the four most prominent seeding countries in that order (Figure 3D, Table S2). From the data, an inferred seeding hierarchy would begin with China at the epicenter of an E-SE Asian influenza subnetwork. Our analysis supports China as the most predictive seeder of many Asian countries, including Hong Kong. Both China and Hong Kong then serve as a launching pad for the dispersal of new seasonal variants to the rest of the world [14], [17], in particular USA and an Oceanic subnetwork dominated by interchange between Australia and New Zealand. Viruses from USA, the largest seeder of the entire world, then spread to a number of South American, European, and African countries. Interestingly, Australia and Hong Kong are equally probable seeders of the USA (Figure 3D). Detailed transmission events are enumerated in Table S2. An inset of the Asian subnetwork is depicted in Figure 4C, a demonstration of this study's high geographic resolution.

### High Circulation between Tropical and Asian Countries with Minimal Local Persistence

As can be seen with the world map plots (Figure 4A,B), a natural representation of the global influenza network is a directed graph with each node representing a clustered region (climate, continent, and country) and each edge representing a seeding event with a weight equal to the number of significant seeding seasons. To quantify observed patterns, we employed principles of graph theory to measure the importance of nodes using four different metrics.

**Figure 6:**
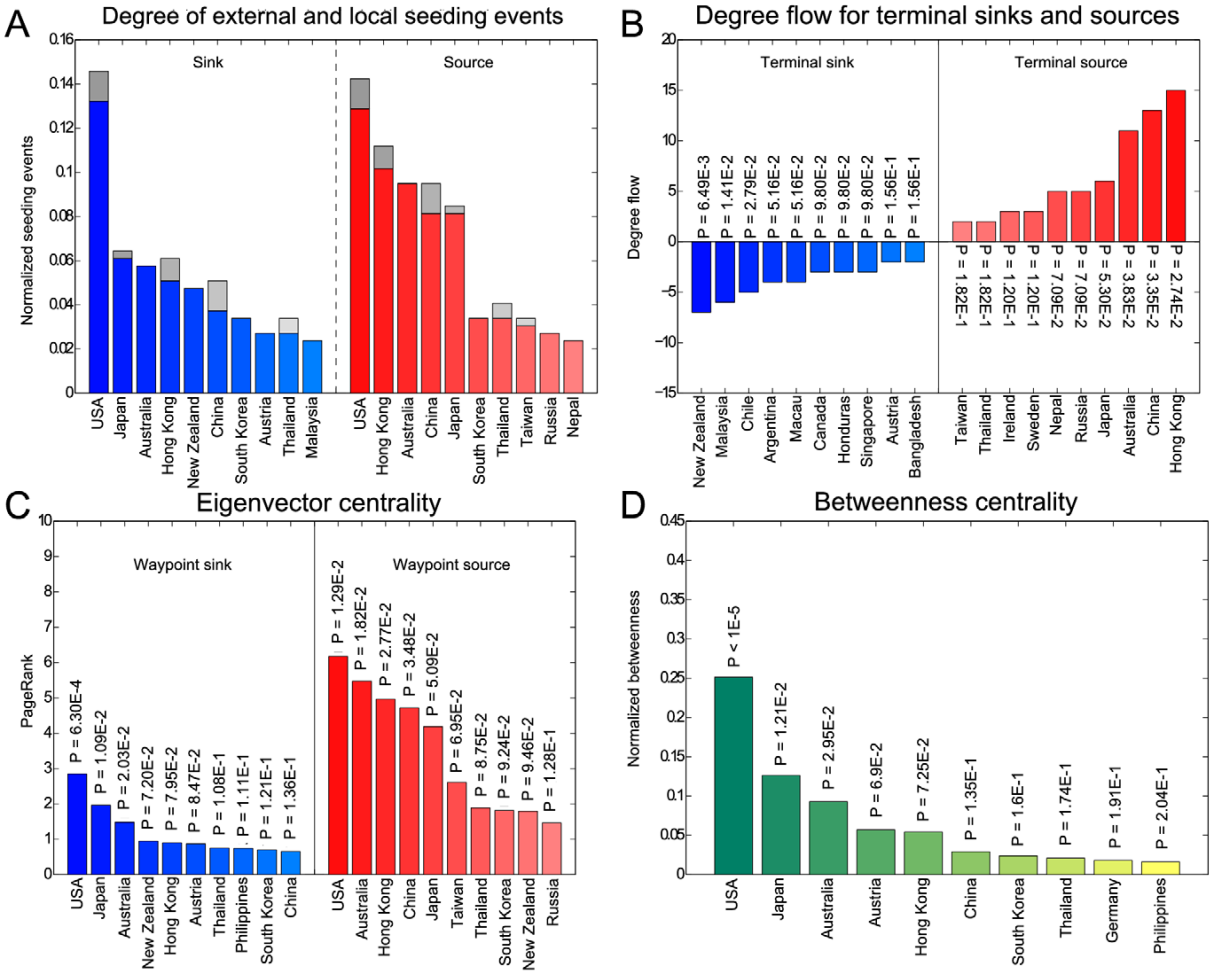
Graph theory metrics of significant seeding (left) continents and (right) countries for H3N2. (A) Indegree and outdegree represent the total number of seeding events into and out of a region, respectively. Internal seeding events (gray bars) play a minor role in overall seeding except in Asia. (B) Terminal sinks/sources: Degree flow measures the difference between seeding events out of and into a node. (C) Waypoint sinks/sources: PageRank categorizes nodes based on the number and quality of links pointing into and out of that node. (D) Betweenness measures the number of shortest paths in a network passing through a given node.

By counting the number of indegrees and outdegrees of each node for H3N2, we identified that the tropics and the northern temperate zone (Figure S4A), specifically Asia and North America (Figure Figure S5A), transmit and receive the most seeding events to and from the rest of the world, respectively. In a similar manner, we identified USA, Hong Kong, Australia, and China as the greatest seeders, and USA, Japan, Australia, and Hong Kong as the most seeded (Figure 6A).

In this analysis, we differentiated between internal (self-seeding) and external (seeding between nodes) transmission events. Importantly, we can accurately detect internal events in temperate countries since their flu seasons are discrete. On the other hand, the specificity for internal events in the tropics is much lower due to unpronounced seasonal peaks. To minimize the number of local false positives, we demarcated seasons within the tropics on a per country basis. We found that for all climate zones except the tropics (Figure S4A) and all continents except Asia (Figure S5A), the number of internal seeding events paled in comparison to the proportion of external seeding events,. The more numerous internal events in the tropics and Asia indicate a high level of circulation between tropical countries and between Asian countries. This pattern is supported by the highly interconnected E-SE Asian subnetwork depicted in Figure 4C. The small proportion of internal events for countries supports the notion that local persistence often plays only a minor role in influenza transmission [8], [9], [10] (Figure 6A).

### Sinks and Sources within the Global Flu Network

Beyond the absolute number of seeding events, a region's influence on global viral spread is also dependent on the topological structure of the graph itself. As an analogy, consider the influenza network as a system of connected train stations each representing a single region seeding influenza. In such systems, trains begin and end their routes at terminal stations. Similarly, influenza commuters begin their journeys at terminal sources and end at terminal sinks in each season. These start and end terminals can represent regions where new influenza variants respectively originate and ultimately spread to. To quantify the terminal characteristic, we calculated the outdegree minus the indegree of each node, which we term “degree flow.” Positive degree flow indicates terminal sources, while negative indicates terminal sinks. Countries were also ranked by calculating the proportion of nodes in a 1,000 randomized networks with a greater, or lesser, degree flow (Text S1).

For analysis by climate zone, the tropics was identified as the only terminal source, suggesting that flu spreads from the tropical belt outward to both temperate zones (Figure S4B). As for continental clustering, Asia was the only terminal source, indicating that global circulation begins in Asia and ends in terminal sink continents, of which North America was the most prominent (Figure S5B). On a country level, Hong Kong and China were the greatest terminal sources, corroborating our observations (Figure 3D). Australia was also a conspicuous terminal source, especially within the Oceanic subnetwork where it seeded the greatest terminal sink, New Zealand. Several South American countries, including Chile and Argentina, figure as terminal sinks too, correlating with such countries as antigenically delayed [8] (Figure 6B).

Trains also stop at waypoint stations, which can be the junction of a large number of routes. Correspondingly, certain regions act as waypoint sources: important intermediate launch pads to other destinations. Others act as waypoint sinks: important points of convergence for multiple routes. Eigenvector centrality can gauge this property on the principle that connections to high-scoring nodes contribute more to the score of the node in question than equivalent connections to low-scoring nodes. We used a method akin to PageRank, Google's method of assigning importance to web pages [25].

Using this method, the northern temperate zone was the most important waypoint source and sink (Figure S4C). Similarly, the predominantly northern temperate continents of North America and Europe were identified as prominent waypoint sources and sinks. Asia, however, was the greatest waypoint source but a poor waypoint sink, correlating with its role as a greater terminal source than North America or Europe (Figure S5C). Interestingly, USA was both the greatest waypoint source and sink (Figure 6C).

H1N1 NA clustering by climate zone produced results similar to that of H3N2 NA. The tropics consistently scored highest by seeding outdegree, positive degree flow, and PageRank source. In addition, the tropics possessed a large amount of internal seeding events. These results emphasize that similar to H3N2, H1N1 circulates within the tropics across seasons only to spread eventually to the temperate zones.

### Disrupting the Global Flow of Influenza

Betweenness measures the number of shortest paths between any two vertices in a network that lie on a given node. In the context of influenza, increasing vaccinations in regions of high betweenness would hypothetically have the greatest effect on diminishing the spread of infection worldwide. This novel strategy contrasts with previous studies simulating containment only at the source of influenza [26], [27]. For H3N2, this criteria highlighted Europe and North America as promising candidates for vaccination programs (Figure S5D). Clustering by country revealed USA, Japan, and Australia as sites in the influenza network vulnerable to disruption (Figure 6D).

## Discussion

Using statistical and network theory analysis, we analyzed H3N2 and H1N1 sequence data to determine the global spread of influenza. Our novel method employs two main strategies to eliminate geographic and seasonal bias: 1) Spatiotemporal clustering of sequence data to count seeding events between clusters and 2) Use of binomial prior probabilities based on the regional proportion of viral isolates to screen for significant seeding events.

Applying these techniques to coding HA and NA segments of H3N2 by climate zone and continent revealed a seeding pattern stemming from the tropics, particularly Asia. HA1 analysis produced a more detailed picture: each year, a wave of seasonal flu originates in China to feed an E-SE Asian subnetwork. From there, China and Hong Kong seed two major subnetworks, each dominated by Australia and USA.

Similar clustering of H1N1 NA sequences by climate zone reproduced tropical transmission to the rest of the world. However, due to inadequate geographic coverage, clustering H1N1 by continent and country proved inconclusive with few significant seeding events detected. One explanation for these results is that important seeding countries, such as China and Hong Kong, were too underrepresented in the dataset. Alternatively, global patterns may be weaker for H1N1 due to cross-reactivity between the two strains [18], [19], a conclusion reflected by the smaller number of seeding events for the strain.

In our analysis, the total number of seeding seasons for each region did not necessarily correspond to the total number of isolates from each region, indicating that our methodology counters data bias. However, certain confounders may affect results. First, selection bias in sampling remarkable variants, such as patients suffering severe rather than mild or non-symptomatic influenza, would poorly represent flu in the general population. Moreover, many sequences had to be excluded from our dataset due to poor annotation and lack of date information. Finally, although our probabilistic methodology accepts regional and temporal variability, it has low sensitivity for detecting anything but particularly significant seeding events for regions with very few sequences. This issue becomes important in analyses with regions that have no sequences whatsoever, as with near-absent sequences from Hong Kong and China for H1N1 HA. The persistence of such bias highlights the continuing need to sequence viruses in underrepresented areas, especially the tropics.

Each year, the current influenza vaccine is formulated separately for the Northern and Southern Hemisphere; one can surmise that two viral strains may not be enough to represent the entire pool of influenza strains around the world. Although there are many other economic and political concerns to consider, our methodology suggests several ways of guiding vaccine strain selection based on biological and epidemiological principles. Graph theory metrics—terminal and waypoint sinks and sources, as well as degree and betweenness centralities—pinpoint potential regions in which increased vaccinations could stem the transmission of influenza globally as well as locally. Increased analytical resolution could optimize vaccine design by choosing the dominant antigenic strain of a country's most predictive seeder. Vaccines could be catered to each country, rather than each hemisphere. At the very least, our analysis advises strain selection from the tropics, from which seasonal strains are dispersed each year. On the other hand, local strain selection within a country should prove comparatively ineffective, as few viruses persist in the inter-epidemic period to seed the following flu season.

Our analysis of terminal sources resonates with an old hypothesis that in southern China, zoonotic infection from live-animals markets [28] selling in particular duck—a natural host of influenza [29]—combined with a dense population for sustained viral circulation, could be the main ingredients for the creation of new seasonal influenza variants. In support, two major acute respiratory infections—SARS [30] and H5N1/97 [31], [32]—have been definitively traced back to southern China, with Hong Kong serving as an important sentinel post for the rest of the world. Other influenza pandemics, 1968 H3N2 (Hong Kong) [28] and even as early as 1889 pandemic influenza [33], have suspected origins in southern China.

It would be interesting to dissect the factors that govern waypoint sources and sinks. For example, air travel and other transportation may play a major role in the dispersal of virus worldwide [8], [19], [34], [35]. Many important hubs of the global flu network, including USA, Australia, Hong Kong, and China, have several of the world's busiest airports [36]. Understanding the reasons for these seeding patterns may offer other strategies for arresting the movement of flu.

The advent of 2009 pandemic S-OIV has largely depleted the number of seasonal H3N2 and H1N1 infections, most likely via cross-reactivity between novel and seasonal strains [22]. Consequently, the conclusions of this paper may not necessarily apply to current dynamics of seasonal H3N2 and H1N1. However, the fact that H1N1 shares a tropic-centric movement pattern with H3N2 despite cross-reactivity suggests that these patterns may still persist even in the presence of the cross-reactive S-OIV. Moreover, this paper demonstrates that when more sequence data is deposited in NCBI, a similar methodology can be applied to predict global circulation of S-OIV as well.

## Materials and Methods

### Data

All sequence data used in this study was publicly available from the National Center for Biotechnology Information database (NCBI) [37]. For each segment, only protein coding regions were considered. Furthermore, we only used sequences with full date (year, month and day) and location information to build hierarchies. Geographical coordinates of each isolate were obtained using geolocation information from Google Maps. Sequences were then aligned using the ClustalW v. 1.83 multiple sequence alignment package using default parameters for H3N2 and H1N1, respectively. For each segment, sequences were aligned and those that were poorly aligned compared to the rest of the dataset were removed until all sequences aligned with a Hamming distance no greater than 0.15. Given estimated mutation rates of 6.7×10^−3^ nucleotide substitutions per site per year [12], [19], Hamming distances over the 20-year span of our dataset are expected to be no more than 0.15 of the sequence length. Outlying sequences were most likely incorrectly sequenced and were discarded from analysis.

### Spatiotemporal Clustering

Our methodology aimed to minimize data bias from geospatial and temporal variability in sequences from NCBI. First, we determined the most parsimonious evolutionary paths traversed by the flu virus. To this end, we sorted sequences from earliest to most recent viral isolates. Working backwards from newest to oldest, we calculated the sequence similarity of each virus to all earlier isolates regardless of geography. We defined a virus's most likely ancestor to be the sequence with minimum Hamming distance. From this data we built evolutionary paths for each virus. Related sequences were clustered (grouped) together by common geography and season to simplify the paths. For example, a chain of related viruses in the same region and season would be collapsed into a single umbrella node representing all of them. Our analysis was then based on looking at the transitions between clusters rather than individual viruses. We counted these “seeding events,” where the closest ancestor of a given cluster of sequences is from a different region or season [8] (Figure 1). When tallying seeding events, non-unique solutions were not considered where a given viral isolate possessed multiple closest ancestors from different geographical zones or seasons (Text S1, Figure S6).

### Modeling Transmission as a Binomial Process

The observed frequencies of seeding events between clusters were compared to expected frequencies based on the prior probability of randomly choosing a sequence from a given geographical zone in the past. Using the binomial distribution with the proportion of prior NCBI sequences as a binomial probability, a p-value was calculated for observing more seeding events than expected. The best predictor of a seeding region for each season had the greatest ratio of observed to expected seeding events with a p-value smaller than 0.05 (Figure 2).

## Supporting Information

Figure S1 Number of H3N2 and H1N1 sequences from the NCBI Influenza Virus Resource sampled from (A) each climate zone and (B) each continent for complete coding segments and the HA1 domain. HA, HA1, and NA possess the greatest geographic coverage of sequences.
(0.61 MB EPS)

Figure S2 Distribution of top ten countries of isolation for NA, HA, and HA1 sequences of H3N2 and H1N1.
(0.26 MB TIF)

Figure S3 Clustering the complete NA coding sequences of H3N2 by (A) climate zone (B) continent, and (C) country.
(0.80 MB EPS)

Figure S4 Rankings of significant seeding and seeded climate zones for H3N2 and H1N1 using different graph theory metrics. (A) The indegree and outdegree of a node represent the total number of seeding events into and out of a region, respectively. Local seeding events depicted in gray play little role in overall seeding except in the tropics. (B) Degree flow measures the difference between seeding events out of and into a node and determines whether it is a terminal sink or source. (C) PageRank uses an algorithm similar to that employed by Google to categorize nodes based on the number and quality of links pointing to that node.
(0.88 MB EPS)

Figure S5 Rankings of significant seeding and seeded continents for H3N2 using different graph theory metrics. (A) The indegree and outdegree of a node represent the total number of seeding events into and out of a region, respectively. Local seeding events depicted in gray play little role in overall seeding except in Asia. (B) Degree flow measures the difference between seeding events out of and into a node and determines whether it is a terminal sink or source. (C) PageRank uses an algorithm similar to that employed by Google to categorize nodes based on the number and quality of links pointing to that node. (D) Betweenness measures the number of shortest paths in a network passing through a given node.
(0.63 MB EPS)

Figure S6 Non-unique solutions per segment for H3N2 and H1N1 clustering by (A) climate zone, (B) continent, and (C) country. The number of these non-unique solutions increases with sequence length, conservation, and geographic coverage. Due to greater genetic diversity, H1N1 has fewer non-unique solutions in all segments apart from M1 and M2.
(0.31 MB EPS)

Table S1 Timing of tropical flu seasons used in the dataset. This data was used to create a consensus tropical season for clustering by climate zone, starting from October 1st to September 30th of the next year. For clustering by country, a unique season was assigned to each tropical country that encompasses both the annual and semi-annual peaks.
(0.07 MB DOC)

Table S2 Top seeding countries after clustering by country for the H3N2 HA1 domain. A distinction is made between externally and locally seeding countries. Note that the total number of significant seeding events does not necessarily correlate with the number of sequences used in the dataset.
(0.30 MB DOC)

Text S1 Detailed description of the methodology, including evaluation of clustering, determining flu seasons, timing of observed seeding events, and network randomization. Detailed description of the methodology, including evaluation of clustering, determining flu seasons, timing of observed seeding events, and network randomization.
(0.02 MB DOCX)
