# Supplementary figures and images for: Network Analysis of Global Influenza Spread
Source: PLoS Comput Biol. 2010 Nov 18;6(11):e1001005. doi: 10.1371/journal.pcbi.1001005 (PMC2987833; doi:10.1371/journal.pcbi.1001005)

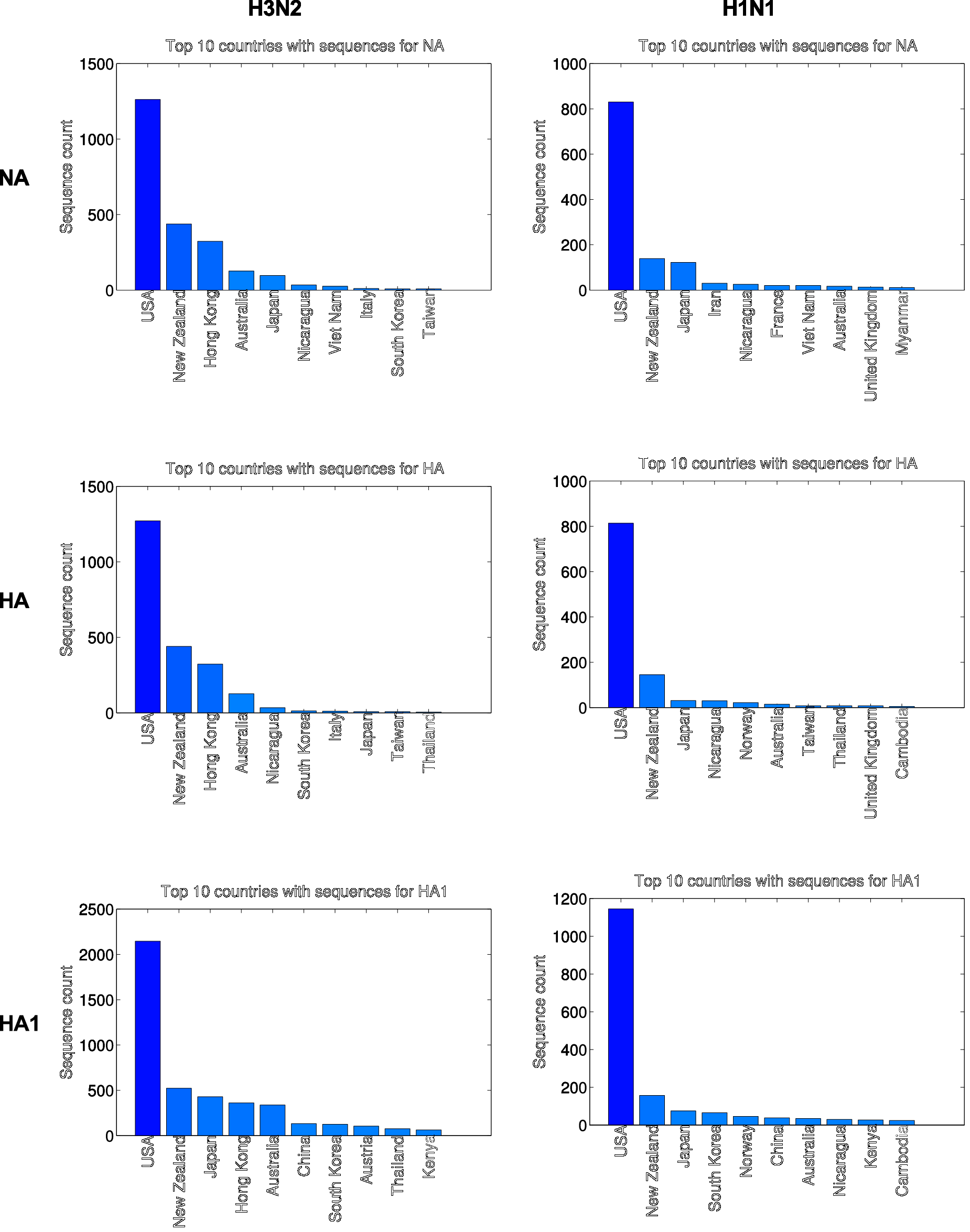

Supplement: Figure S2 — Distribution of top ten countries of isolation for NA, HA, and HA1 sequences of H3N2 and H1N1. (0.26 MB TIF) [file pcbi.1001005.s002.tif]
