# Supplementary material for: Network Analysis of Global Influenza Spread
Source: PLoS Comput Biol. 2010 Nov 18;6(11):e1001005. doi: 10.1371/journal.pcbi.1001005 (PMC2987833; doi:10.1371/journal.pcbi.1001005)
Supplement: Table S1 — Timing of tropical flu seasons used in the dataset. This data was used to create a consensus tropical season for clustering by climate zone, starting from October 1st to September 30th of the next year. For clustering by country, a unique season was assigned to each tropical country that encompasses both the annual and semi-annual peaks. (0.07 MB DOC) [file pcbi.1001005.s007.doc]

Table S1.

| **Country** | **Annual Peak** | **Semi-Annual Peak** | **References** |
| --- | --- | --- | --- |
| **Brazil** | Jun | Jan | (13-14) |
| **Cambodia** | Jun-Dec | - | (15-16) |
| **Guam** | Sept |  | (17) |
| **Hong Kong** | Jan-Feb | Jul-Aug | (18-21) |
| **India** | Jul-Aug | - | (7, 22) |
| **Indonesia** | Dec-Jan | - | (23) |
| **Madagascar** | Jul-Aug | - | (24) |
| **Malaysia** | Jun-Jul | - | (25) |
| **New Caledonia** | Feb-Jun (H3N2), May-Sept (H1N1) | - | (26) |
| **Nicaragua** | Jun-Jul | Nov-Dec | (27) |
| **Philippines** | Jul-Sep | Dec-Jan | (7) |
| **Singapore** | Apr-Jun | Nov-Jan | (7) |
| **Thailand** | Jun-Sep | Jan-Mar | (7, 28) |
| **Vietnam** | May-Sep | - | (7) |
