# Supplementary material for: Network Analysis of Global Influenza Spread
Source: PLoS Comput Biol. 2010 Nov 18;6(11):e1001005. doi: 10.1371/journal.pcbi.1001005 (PMC2987833; doi:10.1371/journal.pcbi.1001005)
Supplement: Table S2 — Top seeding countries after clustering by country for the H3N2 HA1 domain. A distinction is made between externally and locally seeding countries. Note that the total number of significant seeding events does not necessarily correlate with the number of sequences used in the dataset. (0.30 MB DOC) [file pcbi.1001005.s008.doc]

Table S2.

| **Seeding Country** | **Seeded Countries** | **Is Top Seeder** | **Is Top External Seeder** | **Significant Seeding Events Per Country** | **Total Number of Significant Seeding Events** | **Number of Sequences** |
| --- | --- | --- | --- | --- | --- | --- |
| **USA** | USA | * | - | 4 | 40 | 2146 |
|  | Argentina | * | * | 3 |  |  |
|  | Japan | - | - | 2 |  |  |
|  | Kenya | * | * | 2 |  |  |
|  | New Zealand | - | - | 2 |  |  |
|  | Norway | * | * | 2 |  |  |
|  | Austria | * | * | 1 |  |  |
|  | Bangladesh | * | * | 1 |  |  |
|  | Brazil | * | * | 1 |  |  |
|  | Bulgaria | * | * | 1 |  |  |
|  | Chile | - | - | 1 |  |  |
|  | China | - | - | 1 |  |  |
|  | Ecuador | * | * | 1 |  |  |
|  | Egypt | * | * | 1 |  |  |
|  | Germany | * | * | 1 |  |  |
|  | Honduras | * | * | 1 |  |  |
|  | Italy | * | * | 1 |  |  |
|  | Kuwait | * | * | 1 |  |  |
|  | Malaysia | - | - | 1 |  |  |
|  | Mexico | * | * | 1 |  |  |
|  | Nepal | * | * | 1 |  |  |
|  | Netherlands | * | * | 1 |  |  |
|  | New Caledonia | * | * | 1 |  |  |
|  | Northern Mariana Islands | * | * | 1 |  |  |
|  | Philippines | - | - | 1 |  |  |
|  | Qatar | * | * | 1 |  |  |
|  | Russia | * | * | 1 |  |  |
|  | Sri Lanka | * | * | 1 |  |  |
|  | Ukraine | * | * | 1 |  |  |
|  | Uruguay | * | * | 1 |  |  |
|  | Venezuela | * | * | 1 |  |  |
| **Hong Kong** | Hong Kong | * | - | 8 | 37 | 361 |
|  | Australia | - | - | 3 |  |  |
|  | China | - | - | 3 |  |  |
|  | Malaysia | * | * | 3 |  |  |
|  | Singapore | * | * | 3 |  |  |
|  | USA | - | - | 3 |  |  |
|  | Chile | * | * | 2 |  |  |
|  | Philippines | * | * | 2 |  |  |
|  | Thailand | * | * | 2 |  |  |
|  | Finland | * | * | 1 |  |  |
|  | Japan | - | - | 1 |  |  |
|  | New Caledonia | * | * | 1 |  |  |
|  | Northern Mariana Islands | * | * | 1 |  |  |
|  | Norway | - | - | 1 |  |  |
|  | Peru | * | * | 1 |  |  |
|  | South Africa | * | * | 1 |  |  |
|  | Taiwan | - | - | 1 |  |  |
| **China** | Hong Kong | - | * | 5 | 27 | 133 |
|  | China | * | - | 4 |  |  |
|  | USA | * | * | 4 |  |  |
|  | South Korea | - | * | 3 |  |  |
|  | Taiwan | * | * | 2 |  |  |
|  | Argentina | - | - | 1 |  |  |
|  | Australia | - | - | 1 |  |  |
|  | Canada | * | * | 1 |  |  |
|  | Denmark | * | * | 1 |  |  |
|  | Indonesia | * | * | 1 |  |  |
|  | Japan | - | - | 1 |  |  |
|  | New Zealand | - | - | 1 |  |  |
|  | Thailand | - | - | 1 |  |  |
|  | United Kingdom | * | * | 1 |  |  |
| **Australia** | USA | * | * | 4 | 25 | 339 |
|  | France | * | * | 2 |  |  |
|  | Hong Kong | - | - | 2 |  |  |
|  | New Zealand | - | * | 2 |  |  |
|  | Singapore | - | - | 2 |  |  |
|  | Austria | * | * | 1 |  |  |
|  | Canada | * | * | 1 |  |  |
|  | Czech Republic | * | * | 1 |  |  |
|  | Egypt | * | * | 1 |  |  |
|  | Ireland | * | * | 1 |  |  |
|  | Malaysia | - | - | 1 |  |  |
|  | New Caledonia | * | * | 1 |  |  |
|  | Philippines | - | - | 1 |  |  |
|  | Spain | * | * | 1 |  |  |
|  | Sweden | * | * | 1 |  |  |
|  | Taiwan | - | - | 1 |  |  |
|  | Turkey | * | * | 1 |  |  |
|  | United Kingdom | * | * | 1 |  |  |
| **Japan** | China | - | - | 2 | 24 | 429 |
|  | Taiwan | * | * | 2 |  |  |
|  | Argentina | - | - | 1 |  |  |
|  | Australia | - | - | 1 |  |  |
|  | Austria | * | * | 1 |  |  |
|  | Canada | * | * | 1 |  |  |
|  | Chile | - | - | 1 |  |  |
|  | Germany | * | * | 1 |  |  |
|  | Honduras | * | * | 1 |  |  |
|  | Hungary | * | * | 1 |  |  |
|  | India | * | * | 1 |  |  |
|  | Japan | - | - | 1 |  |  |
|  | Macau | * | * | 1 |  |  |
|  | Mongolia | * | * | 1 |  |  |
|  | Paraguay | * | * | 1 |  |  |
|  | Peru | * | * | 1 |  |  |
|  | Russia | * | * | 1 |  |  |
|  | Solomon Islands | * | * | 1 |  |  |
|  | South Korea | - | - | 1 |  |  |
|  | Sri Lanka | * | * | 1 |  |  |
|  | USA | - | - | 1 |  |  |
|  | Viet Nam | * | * | 1 |  |  |
| **South Korea** | Japan | * | * | 4 | 14 | 126 |
|  | South Korea | * | - | 4 |  |  |
|  | USA | - | - | 3 |  |  |
|  | French Guiana | * | * | 1 |  |  |
|  | Guatemala | * | * | 1 |  |  |
|  | Hong Kong | - | - | 1 |  |  |
| **New Zealand** | New Zealand | * | - | 6 | 13 | 523 |
|  | Australia | * | * | 4 |  |  |
|  | Brazil | * | * | 1 |  |  |
|  | Bulgaria | * | * | 1 |  |  |
|  | India | * | * | 1 |  |  |
| **Taiwan** | China | - | - | 3 | 13 | 31 |
|  | Australia | - | - | 1 |  |  |
|  | Germany | * | * | 1 |  |  |
|  | Hong Kong | - | - | 1 |  |  |
|  | Macau | * | * | 1 |  |  |
|  | Nepal | * | * | 1 |  |  |
|  | New Zealand | - | - | 1 |  |  |
|  | Singapore | - | - | 1 |  |  |
|  | Taiwan | - | - | 1 |  |  |
|  | Thailand | - | - | 1 |  |  |
|  | USA | - | - | 1 |  |  |
| **Thailand** | Australia | - | - | 2 | 11 | 76 |
|  | Japan | - | - | 2 |  |  |
|  | Thailand | * | - | 2 |  |  |
|  | Bangladesh | * | * | 1 |  |  |
|  | Canada | * | * | 1 |  |  |
|  | Hong Kong | - | - | 1 |  |  |
|  | New Zealand | - | - | 1 |  |  |
|  | South Korea | - | - | 1 |  |  |
| **Russia** | Australia | - | - | 1 | 8 | 17 |
|  | Austria | * | * | 1 |  |  |
|  | France | - | - | 1 |  |  |
|  | Malaysia | - | - | 1 |  |  |
|  | Saudi Arabia | * | * | 1 |  |  |
|  | Taiwan | - | - | 1 |  |  |
|  | USA | - | - | 1 |  |  |
|  | Ukraine | * | * | 1 |  |  |
| **Nepal** | China | - | - | 1 | 7 | 14 |
|  | Hong Kong | - | - | 1 |  |  |
|  | Hungary | * | * | 1 |  |  |
|  | Japan | - | - | 1 |  |  |
|  | Malaysia | - | - | 1 |  |  |
|  | Thailand | - | - | 1 |  |  |
|  | USA | - | - | 1 |  |  |
| **Norway** | USA | * | * | 4 | 7 | 14 |
|  | New Zealand | - | - | 1 |  |  |
|  | Peru | * | * | 1 |  |  |
|  | Philippines | - | - | 1 |  |  |
| **Austria** | Greece | * | * | 1 | 6 | 105 |
|  | Iceland | * | * | 1 |  |  |
|  | Italy | * | * | 1 |  |  |
|  | Japan | - | - | 1 |  |  |
|  | Mexico | * | * | 1 |  |  |
|  | USA | - | - | 1 |  |  |
| **Germany** | USA | - | - | 2 | 6 | 31 |
|  | Australia | - | - | 1 |  |  |
|  | Japan | - | - | 1 |  |  |
|  | Norway | - | - | 1 |  |  |
|  | Thailand | - | - | 1 |  |  |
| **Philippines** | USA | - | - | 2 | 6 | 20 |
|  | Australia | - | - | 1 |  |  |
|  | Canada | * | * | 1 |  |  |
|  | New Zealand | - | - | 1 |  |  |
|  | Singapore | - | - | 1 |  |  |
| **Brazil** | Brazil | * | - | 1 | 5 | 14 |
|  | Chile | - | - | 1 |  |  |
|  | China | - | - | 1 |  |  |
|  | New Zealand | - | - | 1 |  |  |
|  | USA | - | - | 1 |  |  |
| **Singapore** | Japan | - | - | 1 | 5 | 32 |
|  | New Zealand | - | - | 1 |  |  |
|  | Singapore | - | - | 1 |  |  |
|  | South Korea | - | - | 1 |  |  |
|  | Thailand | - | - | 1 |  |  |
| **France** | Austria | * | * | 1 | 4 | 25 |
|  | Hong Kong | - | - | 1 |  |  |
|  | Thailand | - | - | 1 |  |  |
|  | USA | - | - | 1 |  |  |
| **Ireland** | Australia | - | - | 1 | 4 | 3 |
|  | Norway | - | - | 1 |  |  |
|  | Russia | * | * | 1 |  |  |
|  | USA | - | - | 1 |  |  |
| **Sweden** | France | - | - | 1 | 4 | 5 |
|  | French Guiana | * | * | 1 |  |  |
|  | Romania | * | * | 1 |  |  |
|  | USA | - | - | 1 |  |  |
| **Italy** | Italy | * | - | 1 | 3 | 15 |
|  | Latvia | * | * | 1 |  |  |
|  | USA | - | - | 1 |  |  |
| **Kenya** | Kenya | * | - | 2 | 3 | 63 |
|  | USA | - | - | 1 |  |  |
| **Algeria** | Norway | - | - | 1 | 2 | 1 |
|  | USA | - | - | 1 |  |  |
| **Canada** | Macau | * | * | 1 | 2 | 16 |
|  | USA | - | - | 1 |  |  |
| **Croatia** | Australia | - | - | 1 | 2 | 1 |
|  | Madagascar | * | * | 1 |  |  |
| **Kuwait** | Iraq | * | * | 1 | 2 | 16 |
|  | USA | - | - | 1 |  |  |
| **Latvia** | Austria | * | * | 1 | 2 | 3 |
|  | Romania | * | * | 1 |  |  |
| **Mexico** | France | - | - | 1 | 2 | 12 |
|  | Honduras | * | * | 1 |  |  |
| **Peru** | Germany | * | * | 1 | 2 | 5 |
|  | USA | - | - | 1 |  |  |
| **Switzerland** | Hong Kong | - | - | 1 | 2 | 1 |
|  | South Africa | * | * | 1 |  |  |
| **United Kingdom** | Italy | * | * | 1 | 2 | 15 |
|  | South Korea | - | - | 1 |  |  |
| **Argentina** | Philippines | - | - | 1 | 1 | 16 |
| **Bulgaria** | Japan | - | - | 1 | 1 | 4 |
| **Fiji** | Macau | * | * | 1 | 1 | 1 |
| **Guam** | Japan | - | - | 1 | 1 | 3 |
| **Malaysia** | Japan | - | - | 1 | 1 | 26 |
| **Mongolia** | Japan | - | - | 1 | 1 | 7 |
| **New Caledonia** | Australia | - | - | 1 | 1 | 17 |
| **Nicaragua** | USA | - | - | 1 | 1 | 34 |
| **Northern Mariana Islands** | USA | - | - | 1 | 1 | 5 |
| **Slovenia** | Austria | * | * | 1 | 1 | 1 |
| **Spain** | Austria | * | * | 1 | 1 | 1 |
| **Turkey** | Germany | * | * | 1 | 1 | 1 |
| **Ukraine** | Thailand | - | - | 1 | 1 | 4 |
