# Supplementary material for: Network Analysis of Global Influenza Spread
Source: PLoS Comput Biol. 2010 Nov 18;6(11):e1001005. doi: 10.1371/journal.pcbi.1001005 (PMC2987833; doi:10.1371/journal.pcbi.1001005)
Supplement: Text S1 — Detailed description of the methodology, including evaluation of clustering, determining flu seasons, timing of observed seeding events, and network randomization. Detailed description of the methodology, including evaluation of clustering, determining flu seasons, timing of observed seeding events, and network randomization. (0.02 MB DOCX) [file pcbi.1001005.s009.docx]

**Supplementary Material**

# Evaluation of Clustering

One way of assessing the ability to differentiate variants for a given influenza segment and regional clustering method is to count the non-unique solutions, defined as the instances in which a given isolate has multiple closest ancestors from different geographical zones or seasons. Generally, the number of non-unique solutions is a function of the genetic diversity and the length of a given sequence. For instance, the resolution tends to decrease in highly conserved segments, such as segments M1/M2, and in sequences of smaller length, such as the HA1 domain [1]. Additionally, low geographic or seasonal diversity in a dataset can reduce the number of non-unique solutions by increasing the likelihood that equally close ancestors are from the same location, allowing inference of a unique seeding region and season. For reference, non-unique solutions were measured for all segments of the influenza virus as well as for the HA1 domain (Figure S6). HA and NA incurred only modest numbers of non-unique solutions in both H3N2 and H1N1. Interestingly, H1N1 in general possessed fewer non-unique solutions than H3N2. This observation most likely derives from H1N1’s greater genetic diversity due to fewer seasonal bottlenecks [2].

Most likely due to increased positive selection, the HA1 epitope has the greatest evolutionary rate of mutation in the influenza genome, estimated at 6.7 x 10^–3^ nucleotide substitutions per site per year [1,3]. Resolution of the HA1 domain, however, was comparable to the M1 segment, indicating that the resolution gained from its high evolutionary rate was offset by its greater geographic/seasonal coverage and shorter length, which is half that of the entire HA coding region. Therefore, any discrepancies in findings were resolved in favor of the complete coding sequences of HA and NA over the HA1 domain. Despite the large number of non-unique solutions, we considered any findings from the HA1 domain that were corroborated by the complete coding sequences to be convincing.

# Determining Flu Seasons

The influenza season in the northern and southern temperate zones is well-defined, lasting November to April, and May to September respectively [4]. Defining tropical flu seasons, on the other hand, is particularly difficult since epidemics persist at low levels year-round, often with two ill-defined annual and semi-annual peaks that occur usually, but not always, during the rainy season and the winter season, respectively [5,6,7]. For our analysis, accuracy in defining the endpoints of these seasons is important in order not to falsely increase the number of internal seeding events (a given region seeding itself).

To account for this problem, we compiled from literature a list of all known flu time frames for all tropical countries in our database (Table S1). When clustering by climate zone and continent, we defined a general tropical flu season, from October 1 to September 30^th^ of the next year, to minimize overlap between our defined seasonal endpoints and epidemic peaks. Generalizing tropical flu seasons, however, was not necessary for clustered country analysis; we therefore defined flu seasons specific to each country, beginning before the larger annual peak and ending after the smaller semiannual peak.

# Timing of Observed Seeding Events

In constructing the network of seeding events, we also investigated the time frame between each pair of seeded and seeding sequences. We found that no matter the method of clustering or segment analyzed, the overwhelming majority (>90%) of seeding sequences came from within one year of the seeded sequence. This finding suggested that placing a temporal threshold on which isolates to consider as seeding candidates to be unnecessary.

# Randomization of Networks

As a comparison to experimental data, randomized networks must control for particular epidemiological principles that constrain the data. One philosophical question that such networks must address is to what extent are seeding events independent. One possible argument is that all seeding events are universally independent of each other. This makes sense to a certain extent; an influenza carrier in the northern hemisphere does not have any direct effect on a carrier in the south. Even carriers in the same region and season are independent agents of free will and can make choices to travel to any random place. In the context of our network model, this argument asserts that each unit of a weighted edge represents a seeding event independent of all others, even those in the same edge.

A randomized network that reflects transmission of complete independence would mix seeding events irrespective of their origin or target while preserving the number of seeding events into and out of each region. To this end, we implemented a Markov chain that iteratively samples a pair of seeding regions (x_1_, x_2_) and a pair of seeded regions (y_1_, y_2_) at random. Given that x_i_->y_j_ is the number of seeding events from region x_i_ to region y_i_, the counts for seeding events were randomly mixed such that $\sum_{i} x_{i}\to y_{j}$ and $\sum_{j} x_{i}\to y_{j}$ remain constant. We ran this procedure 10,000 times with a mixing time of 100,000 steps. This methodology is akin to Markov sampling to randomize an NxN contingency table [8].

Of course, complete independence cannot totally describe what occurs in reality. For example, a northern hemisphere carrier of influenza can indirectly suppress a southern carrier’s ability to transmit to the north through herd immunity. Moreover, one could argue that carriers in the same region and season are more likely to travel to the same places due to similar backgrounds. This is even truer if one also considers that available transportation routes may discretize the number of travel destinations from a given location. One can imagine that seeding patterns may largely mirror the topology of the international aviation network [3,9,10,11]. In this way, the seeding events of a weighted edge are interdependent and cannot be fractionized.

A natural way of simulating such local interdependence and interregional independence is to shuffle edges such that topological indegree and outdegree is preserved. In this process, two links at a time, x_1_-y_1_ and x_2_-y_2_, are chosen from the observed graph and rewired to form x_1_-y_2_ and x_2_-y_1_ [12]. We ran this procedure 10,000 times, with 100,000 shuffling iterations. This is a process often used to analyze protein-protein interaction networks.

Our choice for a random network depended on the specific metrics of network centrality we were testing. Degree flow, defined as the difference between seeding events out of and into a region, is invariant to mixing by complete independence. Therefore, we randomized degree flow by rewiring edges. On the other hand, PageRank and betweenness are metrics dependent on topology and were more effectively randomized by mixing the weights of seeding events.

These techniques worked effectively for clustering by continent and country, but were unable to randomize the 3x3 matrix produced by climate zone clustering without mixing internal seeding events along the matrix diagonal. To test only external seeding events, we used a variant of an Erdos-Reyni random graph with preserved number of edges for clustering by climate zone.

One should wonder if, due to the number of countries in HA1, HA, and NA (81, 17, and 21 countries respectively), multiple hypotheses corrections could alter the significance of the results. However, the independent analysis of HA and NA data reveals consistent results, namely an E-SE Asian origin with China and Hong-Kong as the most significant sources. For instance, the p-value of Hong Kong for HA1 data is p = 0.023, p=0.027 for HA and p=0.014 for NA. There are 16 countries in common between HA and NA. A corrected p-value for a country in the intersection of HA and NA datasets is x*(1-log(x)), where x=p1*p2. Consequent Bonferroni correction gives a p-value of p=0.05 with 16 overlapping countries.

# Supporting Figure Legends

Figure S1. Number of H3N2 and H1N1 sequences from the NCBI Influenza Virus Resource sampled from (A) each climate zone and (B) each continent for complete coding segments and the HA1 domain. HA, HA1, and NA possess the greatest geographic coverage of sequences.

Figure S2. Distribution of top ten countries of isolation for NA, HA, and HA1 sequences of H3N2 and H1N1.

Figure S3. Clustering the complete NA coding sequences of H3N2 by (A) climate zone. (B) continent, and (C) country.

Figure S4. Rankings of significant seeding and seeded climate zones for H3N2 and H1N1 using different graph theory metrics. (A) The indegree and outdegree of a node represent the total number of seeding events into and out of a region, respectively. Local seeding events depicted in gray play little role in overall seeding except in the tropics. (B) Degree flow measures the difference between seeding events out of and into a node and determines whether it is a terminal sink or source. (C) PageRank uses an algorithm similar to that employed by Google to categorize nodes based on the number and quality of links pointing to that node.

Figure S5. Rankings of significant seeding and seeded continents for H3N2 using different graph theory metrics. (A) The indegree and outdegree of a node represent the total number of seeding events into and out of a region, respectively. Local seeding events depicted in gray play little role in overall seeding except in Asia. (B) Degree flow measures the difference between seeding events out of and into a node and determines whether it is a terminal sink or source. (C) PageRank uses an algorithm similar to that employed by Google to categorize nodes based on the number and quality of links pointing to that node. (D) Betweenness measures the number of shortest paths in a network passing through a given node.

Figure S6**.** Non-unique solutions per segment for H3N2 and H1N1 clustering by (A) climate zone, (B) continent, and (C) country. The number of these non-unique solutions increases with sequence length, conservation, and geographic coverage. Due to greater genetic diversity, H1N1 has fewer non-unique solutions in all segments apart from M1 and M2.

**Supporting Table Legends**

Table S1. Timing of Tropical Flu Seasons Used in the Dataset. This data was used to create a consensus tropical season for clustering by climate zone, starting from October 1^st^ to September 30^th^ of the next year. For clustering by country, a unique season was assigned to each tropical country that encompasses both the annual and semi-annual peaks.

Table S2. Top seeding countries after clustering by country for the H3N2 HA1 domain. A distinction is made between externally and locally seeding countries. Note that the total number of significant seeding events does not necessarily correlate with the number of sequences used in the dataset.

**Supporting References**

1. McHardy AC, Adams B (2009) The Role of Genomics in Tracking the Evolution of Influenza A Virus. PLoS Pathog 5: e1000566.

2. Rambaut A, Pybus OG, Nelson MI, Viboud C, Taubenberger JK, et al. (2008) The genomic and epidemiological dynamics of human influenza A virus. Nature 453: 615-619.

3. Lofgren E, Fefferman NH, Naumov YN, Gorski J, Naumova EN (2007) Influenza Seasonality: Underlying Causes and Modeling Theories. J Virol 81: 5429-5436.

4. Simonsen L (1999) The global impact of influenza on morbidity and mortality. Vaccine 17: S3-S10.

5. Viboud Cc, Alonso WJ, Simonsen L (2006) Influenza in Tropical Regions. PLoS Med 3: e89.

6. Soebiyanto RP, Adimi F, Kiang RK (2010) Modeling and Predicting Seasonal Influenza Transmission in Warm Regions Using Climatological Parameters. PLoS ONE 5: e9450.

7. Hampson A (1999) Epidemiological data on influenza in Asian countries. Vaccine 17: S19 - S23.

8. Cryan M, M. Dyer, et al. . Rapidly Mixing Markov Chains for Sampling Contingency Tables with a Constant Number of Rows. In: Martin D, Leslie Ann G, Mark J, Russell M, editors; 2002. pp. 711-711.

9. Russell CA, Jones TC, Barr IG, Cox NJ, Garten RJ, et al. (2008) The Global Circulation of Seasonal Influenza A (H3N2) Viruses. Science 320: 340-346.

10. Colizza V, Barrat A, Barthelemy M, Valleron A-J, Vespignani A (2007) Modeling the Worldwide Spread of Pandemic Influenza: Baseline Case and Containment Interventions. PLoS Med 4: e13.

11. Brownstein JS, Wolfe CJ, Mandl KD (2006) Empirical Evidence for the Effect of Airline Travel on Inter-Regional Influenza Spread in the United States. PLoS Med 3: e401.

12. Newman MEJ, Strogatz SH, Watts DJ (2001) Random graphs with arbitrary degree distributions and their applications. Physical Review E 64: 026118.

13. de Arudda E, Hayden F, McAuliffe J, de Souza M, Mota S, et al. (1991) Acute respiratory viral infections in ambulatory children of urban northeast Brazil. J Infect Dis 164: 252 - 258.

14. Alonso W, Viboud C, Simonsen L, Hirano E, Daufenbach L, et al. (2007) Seasonality of influenza in Brasil: a traveling wawe from the Amazon to the subtropics. Am J Epidemiol 165: 1434 - 1442.

15. Buchy P, Mardy S, Vong S, Toyoda T, Miller M, et al. (2007) Influenza A/H5N1 Virus Infection in Humans in Cambodia. J Clin Virol 39: 164 - 168.

16. Mardy S, Ly S, Heng S, Vong S, Huch C, et al. (2009) Influenza activity in Cambodia during 2006-2008. BMC Infectious Diseases 9: 168.

17. Haddock R (1977) Guam's Influenza Experience. AJPH 67.

18. Chiu SS, Lau YL, Chan KH, Wong WHS, Peiris JSM (2002) Influenza-Related Hospitalizations among Children in Hong Kong. N Engl J Med 347: 2097-2103.

19. Wong CM, Yang L, Chan KP, Leung GM, Chan KH, et al. (2006) Influenza-Associated Hospitalization in a Subtropical City. PLoS Med 3: e121.

20. Yang L, Wong CM, Lau EHY, Chan KP, Ou CQ, et al. (2008) Synchrony of Clinical and Laboratory Surveillance for Influenza in Hong Kong. PLoS ONE 3: e1399.

21. Paul KSC, Mok HY, Lee TC, Ida MTC, Wai-Yip L, et al. (2009) Seasonal influenza activity in Hong Kong and its association with meteorological variations. Journal of Medical Virology 81: 1797-1806.

22. Rao B, Banerjee K (1993) Influenza surveillance in Pune, India, 1978-90. Bull World Health Organ 71: 177 - 181.

23. Beckett CG, H. Kosasih, et al. (Aug. 15, 2004) Influenza Surveillance in Indonesia: 1999-2003. Clinical Infectious Diseases 39: 443-449.

24. (2002) WHO-GOARN investigation team. Outbreak of influenza, Madagascar, July-August 2002. Euro Surveill 7: 387.

25. Samad AH, Haji Usul MHB, Zakaria D, Ismail R, Tasset-Tisseau A, et al. (2004) Influenza vaccination in a Malaysian company: what are costs and benefits for the employer? International Congress Series 1263: 585-589.

26. Berlioz-Arthaud A, Barr IG (2005) Laboratory-based influenza surveillance in New Caledonia, 1999-2003. Transactions of the Royal Society of Tropical Medicine and Hygiene 99: 290-300.

27. Gordon A OO, Kuan G, Reingold A, Saborio S, Balmaseda A, et al. (2009 Mar) Prevalence and seasonality of influenza-like illness in children, Nicaragua, 2005–2007. Emerg Infect Dis.

28. Waicharoen S, Thawatsupha P, Chittaganpitch M, Maneewong P, Thanadachakul T, et al. (2008) Influenza viruses circulating in Thailand in 2004 and 2005. Jpn J Infect Dis 61: 321 - 323.
